# Large Language Models as Examinees and Graders in Simulated General Surgery Oral Board-Style Cases: A Psychometric Pilot Study

**DOI:** 10.7759/cureus.113882

**Published:** 2026-08-03

**Authors:** Kian A Huang, Haris K Choudhary, Allan L Xu, Wid Alhassani, David Samvelian, Ronald J Weigel, Marshall S Baker, Fred A Luchette, Paul C Kuo

**Affiliations:** 1 General Surgery, University of South Florida Morsani College of Medicine, Tampa, USA; 2 General Surgery, The University of Iowa, Iowa City, USA; 3 General Surgery, University of Utah Health, Salt Lake City, USA; 4 General Surgery, Loyola University Chicago, Chicago, USA

**Keywords:** artificial intelligence, inter-rater reliability, large language models, oral board examination, psychometrics, surgical education

## Abstract

Introduction: Surgical oral board examinations are vulnerable to examiner variability, potentially compromising scoring reliability. This study evaluated six public frontier large language models (LLMs) as both examinees and graders on general surgery oral board-style cases.

Methods: Six commercially available LLMs were assessed across three standardized cases using a fully crossed psychometric design. Responses were graded on a 0-3 ordinal scale by six LLMs and three blinded senior surgeons using a structured rubric with anchored descriptors for each score level. Analyses included performance comparisons, reliability testing, and variance decomposition.

Results: All LLMs achieved passing or near-passing performance. Significant differences existed between models (Friedman χ² = 12.51, p = 0.028). AI graders demonstrated greater internal consistency than the three-surgeon human panel in this pilot study (Cronbach's α = 0.697 vs. −0.923), though this comparison is limited by the small human panel and the absence of rater calibration for human graders. The dominant source of score variance was the Examinee × Rater interaction, suggesting that grader-driven disagreement accounted for more score variability than actual examinee performance differences in this dataset.

Conclusions: LLMs demonstrate passing-level oral board performance and more consistent grading than human surgeons in this simulation context, suggesting potential value for further investigation of LLM integration into surgical training and assessment frameworks, pending replication in larger samples. Future work should evaluate whether these findings generalize to live examination settings, compare against calibrated human rater panels, and explore AI-augmented panel designs.

## Introduction

The General Surgery Certifying Examination (GSCE) is a virtual oral examination consisting of three consecutive 30-minute sessions, each conducted by a team of two examiners, with the explicit purpose of evaluating a candidate's clinical skills in organizing diagnostic workup and determining appropriate therapy for a broad range of surgical problems [1]. Despite their central role in gatekeeping entry to independent surgical practice, oral board and structured clinical examinations are subject to measurable examiner variability, with empirical studies demonstrating that differences in examiner scoring can significantly influence candidate outcomes and raise concerns regarding the reliability and consistency of assessment decisions, including implications for fairness and validity of pass/fail outcomes [2].

The emergence of large language models (LLMs) as clinically capable systems has been rapid and consequential. Within the span of a few years, AI systems have demonstrated the ability to pass the United States Medical Licensing Examination (USMLE) [3], assist with diagnostic reasoning across medical specialties [4], generate operative plans, and synthesize complex surgical literature within evidence-based decision support frameworks [5]. These capabilities position LLMs as plausible tools for augmenting surgical training, not merely as information retrieval systems, but as interactive assessors capable of simulating the evaluative role of a human examiner. This study was designed to address two foundational questions: whether contemporary LLMs can demonstrate reasoning consistent with passing oral board performance, and whether AI graders exhibit comparable or superior reliability relative to trained human surgeons.

Compounding this reliability problem, traditional oral examination formats provide limited structured feedback, constraining their educational value primarily to summative pass/fail decisions rather than formative skill development. Assessment systems in surgical training, including oral examination formats, have been described as primarily summative, influenced by extraneous examinee characteristics, with limited structured mechanisms for formative feedback and skill development [6,7]. Access to repeated practice opportunities is further influenced by variability in training environments and faculty availability across programs [8]. These structural limitations, variable scoring, limited feedback, and uneven access to preparatory experiences create conditions in which AI-assisted assessment may offer opportunities for greater standardization and scalability.

Prior work on AI in medical education has focused predominantly on written examination formats, particularly multiple-choice assessments analogous to licensing examinations. ChatGPT (OpenAI, Inc., San Francisco, California, USA) has demonstrated performance at or near the USMLE passing threshold on representative question sets without task-specific fine-tuning [3] while Med-PaLM (Google Research, Mountain View, California, USA) has shown that instruction tuning can substantially improve performance on medically oriented question-answering benchmarks compared with base LLMs [9]. Performance on such instruments, while notable, does not address the dynamic, case-based reasoning demanded by oral board formats. 

AI graders offer several properties that may address documented weaknesses of human examiner panels. Human scoring is often distorted by fatigue, anchoring bias, and the halo effect, the latter of which can produce inappropriately uniform ratings across discrete performance domains [10]. In contrast, AI systems are not subject to fatigue, sequential contrast effects, or the examiner-level halo biases documented in human rating panels. They can apply scoring criteria with high fidelity across large volumes of responses and are available without scheduling constraints, enabling scalable and frequent assessment. These properties are particularly relevant in resource-limited and global surgical training environments where access to trained surgical educators and assessment infrastructure may be restricted [11].

Psychometric frameworks, including generalizability theory, intraclass correlation, and Cronbach's alpha, provide rigorous, standardized methods for directly comparing AI and human grader consistency, enabling an empirical rather than speculative evaluation of their relative reliability. Generalizability theory decomposes observed score variance into its underlying sources, such as examinee ability, case difficulty, rater stringency, and their interactions, allowing for a more comprehensive characterization of measurement error than classical reliability coefficients alone [12].

The primary objective of this study was to determine whether contemporary LLMs can achieve passing performance on standardized general surgery oral board-style cases. The secondary objective was to evaluate whether AI graders demonstrate superior reliability compared to human surgical graders, using a fully crossed psychometric design. A tertiary aim was to characterize the primary sources of scoring variance to inform future instrument design and examiner standardization efforts.

## Materials and methods

This study was designed as a prospective, fully crossed psychometric evaluation of LLMs functioning as both examinees and graders in simulated general surgery oral board-style scenarios. The majority of the study was conducted at the home campus at Tampa General Hospital, Florida, USA. Six commercially available frontier LLMs were included and evaluated across three standardized cases involving operative decision-making: ChatGPT 5.1 (GPT-5.1 Instant), Gemini 3 Pro (Google LLC, Mountain View, California, USA), Claude Sonnet 4.5 (Anthropic PBC, San Francisco, California, USA), Grok 4.1 (xAI Corp., San Francisco, California, USA), DeepSeek-V3.2 (DeepSeek, Hangzhou, Zhejiang, China), and OpenEvidence (OpenEvidence, Inc., Miami, Florida, USA) (no version name was available to document, but was utilized from January to March of 2026). All models were accessed via their respective public interfaces during the study period and were evaluated using their default settings. Senior surgeons served as external validation graders and were blinded to model identity and performance. Each model was anonymized during analysis (LLM A-F) and later unblinded for reporting. Models represented leading platforms available at the time of the study and were accessed through standard consumer interfaces without fine-tuning or domain-specific training. All models received identical text-only prompts; no multimodal inputs were used. Model versions were queried using default temperature settings, and no system-level prompt modifications were applied.

Three unique surgical cases were created and inspired based on content from the General Surgery Oral Board Review to reflect the structure and rigor of the American Board of Surgery (ABS) oral examination format and reviewed by senior surgeons for clinical validity [13]. The selection of three cases drawn from common general surgery presentations (ventral hernia, small bowel obstruction, acute appendicitis) was intentional for this proof-of-concept study, but limits generalizability to the full breadth of ABS oral examination content; this constraint is addressed in the Limitations. These cases included: a patient with a symptomatic ventral hernia requiring evaluation and operative planning; a patient with small bowel obstruction requiring diagnostic workup and management decisions; and a patient with suspected acute appendicitis requiring diagnostic reasoning and surgical management. Each case was delivered as a structured prompt designed to simulate the iterative, probing nature of a live oral board examination.

Prior to testing, each LLM examinee was provided with a standardized orientation prompt designed to simulate the dynamics of a real-world oral certifying examination. The full prompt is reproduced below:

'You are about to engage in a Mock ABS Certifying Examination. You are the primary surgeon responsible for managing the patient's surgical needs and complications. You will first prioritize obtaining a specific history and physical examination before considering labs and imaging. No consults from other surgical specialties are allowed. The scenarios will be complex and involve patients with comorbidities or other not straightforward features. You will not be prompted for the next steps or given hints unless you ask for them. Each vignette will begin with the patient's age, sex, chief complaint, and care setting. I will not provide vitals, history, exam findings, labs, or imaging unless you specifically ask for them. When requesting vitals or labs, use a markdown table. You will be asked to describe the preoperative workup, detail the steps of the operation, and discuss post-operative care. You will also be asked how you would manage a possible intraoperative or postoperative complication. Treat this as a dialogue with the examiners, responding promptly and decisively in a step-by-step manner while maintaining a conversational tone. Provide concise reasoning behind your actions, explaining not just the what but also the why. Be clear and concise, tailoring your answers directly to the questions asked. Be authentic in your responses, focusing on patient-centered care, safety, and optimal outcomes. Back up your decisions with logical and evidence-based reasoning. You will be assessed according to the following essential attributes: organized approach and solid rationale; rapid determination of key findings; effective use of clinical knowledge; avoidance of errors; recognition of personal limitations; prompt and flexible response to alterations in the patient's course; and overall demonstration of appropriate surgical judgment, clinical reasoning, and problem-solving ability.'

Before grading, each LLM grader received a standardized orientation prompt, reproduced below, to establish the examination context and scoring expectations:

'You are about to serve as an examiner evaluating responses to a Mock ABS Certifying Examination. You will be assessing the performance of examinees on complex surgical scenarios using a standardized four-point scoring rubric (0 to 3). For each response, you will evaluate the examinee's performance across the following essential attributes of a certifiable surgeon: organized approach and solid rationale; rapid determination and interpretation of key findings; effective and efficient use of clinical knowledge; avoidance of errors and critical fails; recognition of personal limitations; prompt and flexible response to alterations in the patient's course; and overall demonstration of appropriate surgical judgment, clinical reasoning, and problem-solving ability. You will assign a global score from 0 to 3 for each case, with 0 representing a critical fail or inability to manage the case, 1 representing significant deficiencies, 2 representing competent performance with minor omissions, and 3 representing superior performance without significant error. Provide concise justification for each score, referencing specific strengths and weaknesses observed in the response. Be fair, consistent, and evidence-based in your evaluations, focusing on the clinical content and reasoning demonstrated rather than style or presentation. Treat this as you would a real oral examination, applying the same standards expected of a certifying surgeon.'

Responses were graded using a structured four-point ordinal rubric (0-3) with explicitly anchored behavioral descriptors for each score level, developed iteratively with input from senior surgeons and refined to maximize inter-rater clarity. A score of 3 (Pass) was defined as a response that was clinically appropriate, complete, and safe: the examinee correctly prioritized the diagnostic workup, identified the correct operative or management decision, and articulated supporting reasoning consistent with evidence-based surgical practice. A score of 2 (Equivocal) was assigned when the examinee's response was broadly appropriate and safe but incomplete, demonstrating adequate overall management without achieving the specificity or depth expected of a board-eligible surgeon; responses in this category might correctly identify a management strategy but omit critical nuances (e.g., timing of intervention, relevant contraindications, or important adjuncts). A score of 1 (Fail) indicated a response containing significant omissions, incorrect reasoning, or a clinically problematic recommendation that would be unlikely to be endorsed by a senior surgeon, though not rising to the level of immediate patient endangerment. A score of 0 (Critical Fail) was reserved for responses recommending an action with the potential for serious patient harm, such as recommending non-operative management for a perforated viscus or failing to recognize a surgical emergency requiring immediate intervention.

Each grader scored responses independently using a standardized scoring form that displayed the rubric anchors alongside each question; graders were instructed to select the single best descriptor and were not permitted to assign half-point values. All graders, both AI and human, received the same rubric document prior to scoring. For AI graders, rubric instructions were embedded directly within the grading prompt to ensure consistent application across all examinee-case combinations. For human graders, the same rubric was freely available for reference at any point. One additional grader was recruited but was lost to follow-up, did not complete the full rating assignment, and was therefore excluded from the analysis.

Statistical and psychometric analyses were conducted using Python 3.11 (Python Software Foundation, Beaverton, Oregon, USA) with the following libraries: NumPy 1.26, SciPy 1.11, pandas 2.1, and pingouin 0.5 for psychometric analyses. All analysis code is available upon request. Descriptive statistics were calculated for each examinee and grader. Inter-rater reliability was assessed using intraclass correlation coefficients and Krippendorff's alpha. 

Differences in examinee performance were evaluated using the Friedman test with post-hoc pairwise Wilcoxon comparisons adjusted for multiple testing using Bonferroni correction. Generalizability theory was applied using a fully crossed person-by-rater-by-case design to estimate variance components and project reliability under varying panel configurations. Internal consistency was assessed using Cronbach's alpha; pass/fail decision consistency was evaluated across all grader pairs. A sensitivity analysis excluding self-grading observations was performed to assess potential self-serving bias. A significance threshold of α = 0.05 was used throughout.

Because the fully crossed design permitted AI models to grade their responses, a pre-specified sensitivity analysis excluding self-grading observations was conducted as the primary method for assessing self-serving bias; results of this analysis are reported alongside the main findings.

## Results

Examinee performance overview

Examinee performance across all graders and cases is summarized in Table 1. Across 162 graded observations (six LLMs × nine graders × three cases), the overall mean score was 2.51 (SD 0.66) on a 0-3 ordinal scale. Critically, no Critical Fail ratings (score = 0) were assigned by any grader to any examinee, establishing a meaningful safety floor: despite operating without task-specific fine-tuning on surgical oral board formats, no frontier LLM generated catastrophically unsafe or clinically indefensible recommendations across any of the three cases. A total of 14 Fail ratings were observed across the full dataset, corresponding to an overall fail rate of 8.6%, concentrated among the lower-performing models.

**Table 1:** Examinee performance across human graders Descriptive statistics for all six large language models (LLM) examinees based exclusively on scores assigned by the three human surgical graders (Surgeons 1-3). Scores are reported on a 0-3 ordinal scale: 0 = Critical Fail (clinically dangerous response), 1 = Fail (significant omissions or errors), 2 = Equivocal (adequate but incomplete), 3 = Pass (clinically appropriate and complete). Pass% represents the proportion of ratings scored 3; Equivocal% represents the proportion scored 2; Fail% represents the proportion scored 1. No Critical Fail (score = 0) ratings were assigned by any grader to any examinee. ChatGPT 5.1 (OpenAI, Inc., San Francisco, California, USA), Gemini 3 Pro (Google LLC, Mountain View, California, USA), Claude Sonnet 4.5 (Anthropic PBC, San Francisco, California, USA), Grok 4.1 (xAI Corp., San Francisco, California, USA), DeepSeek-V3.2 (DeepSeek, Hangzhou, Zhejiang, China), OpenEvidence (OpenEvidence, Inc., Miami, Florida, USA)

Label	Identity	Mean	SD	95% Confidence Interval	Median	Pass %	Equivocal %	Fail %
LLM F	OpenEvidence	2.89	0.32	(2.76, 3.02)	3	88.90%	11.10%	0.00%
LLM C	DeepSeek V3.2	2.67	0.55	(2.45, 2.89)	3	70.40%	25.90%	3.70%
LLM A	ChatGPT 5.1	2.52	0.58	(2.29, 2.75)	3	55.60%	40.70%	3.70%
LLM B	Sonnet 4.5	2.41	0.75	(2.11, 2.70)	3	55.60%	29.60%	14.80%
LLM D	Gemini 3 Pro	2.3	0.72	(2.01, 2.58)	2	44.40%	40.70%	14.80%
LLM E	Grok 4.1	2.3	0.72	(2.01, 2.58)	2	44.40%	40.70%	14.80%
All	Grand Mean	2.51	0.66	None	None	59.90%	31.50%	8.60%

LLM F (OpenEvidence) demonstrated a mean score of 2.89 (SD 0.32; 95% CI (2.76, 3.02)) and a Fail rate of 0.0%, representing the top of the performance distribution with a confidence interval that does not overlap the equivocal threshold. LLM C (DeepSeek V3.2) achieved a mean of 2.67 (SD 0.55; 95% CI (2.45, 2.89)) and a Fail rate of 3.7%. LLM A (ChatGPT 5.1) demonstrated a mean of 2.52 (SD 0.58; 95% CI (2.29, 2.75)), placing it near the midpoint of the performance distribution with a high equivocal proportion (40.7%), suggesting a pattern of adequate but not fully developed reasoning. LLM B (Sonnet 4.5) achieved a mean of 2.41 (SD 0.75; 95% CI (2.11, 2.70)), with a Fail rate of 14.8% and a wider confidence interval reflecting greater scoring inconsistency across cases or graders. LLM D (Gemini 3 Pro) and LLM E (Grok 4.1) demonstrated statistically identical profiles, mean scores of 2.30 (SD 0.72; 95% CI (2.01, 2.58)) each, with Fail rates of 14.8% and the lowest Pass proportions in the cohort (44.4% each), positioning them at the lower tier of the performance distribution while still remaining above the critical fail threshold. Notably, the confidence intervals for LLM D and LLM E overlap substantially with those of LLM B and LLM A, indicating that performance differences among the four middle-ranked models should be interpreted cautiously. 

Differences in examinee performance across the full panel were statistically significant by the Friedman test (χ² = 12.51, df = 5, p = 0.028), confirming that the observed performance hierarchy does not reflect random variation. Post-hoc pairwise comparisons with Bonferroni correction identified the LLM F vs. LLM D and LLM F vs. LLM E contrasts as the primary drivers of this signal, consistent with the substantial performance gap between OpenEvidence and the lowest-scoring models.

**Figure 1:**
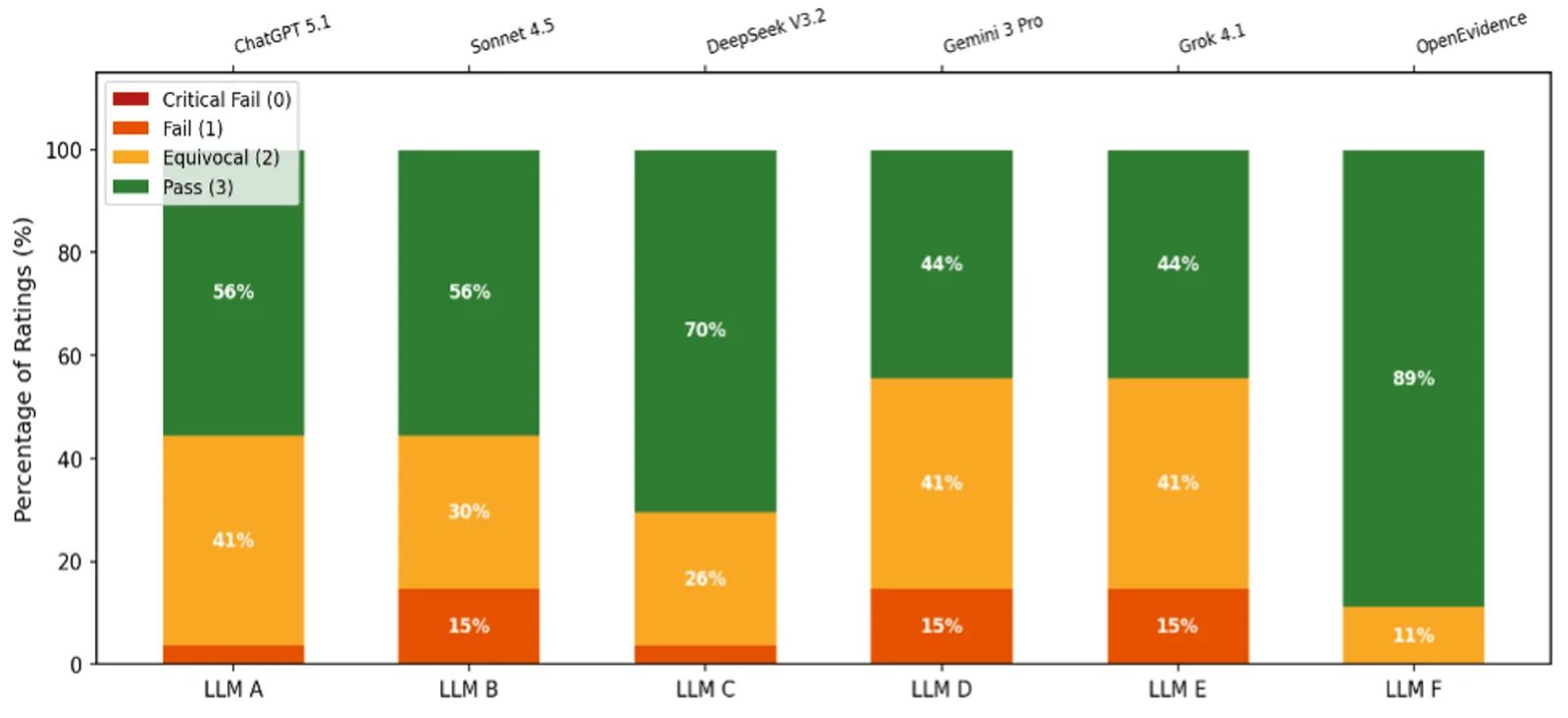
Score distribution by examinee Stacked bar chart illustrating the proportional distribution of categorical rating outcomes, Pass (score = 3, dark green), Equivocal (score = 2, orange-yellow), Fail (score = 1, orange-red), and Critical Fail (score = 0, red), for each of the six large language models (LLM) examinees, aggregated across all nine graders and all three clinical cases (n = 27 ratings per examinee). Model identity labels are displayed above each bar (ChatGPT 5.1, Sonnet 4.5, DeepSeek V3.2, Gemini 3 Pro, Grok 4.1, OpenEvidence). No Critical Fail ratings were assigned by any grader. ChatGPT 5.1 (OpenAI, Inc., San Francisco, California, USA), Gemini 3 Pro (Google LLC, Mountain View, California, USA), Claude Sonnet 4.5 (Anthropic PBC, San Francisco, California, USA), Grok 4.1 (xAI Corp., San Francisco, California, USA), DeepSeek-V3.2 (DeepSeek, Hangzhou, Zhejiang, China), OpenEvidence (OpenEvidence, Inc., Miami, Florida, USA)

The distribution of categorical ratings for each examinee is depicted in Figure 1, which plots the proportional composition of Pass, Equivocal, and Fail ratings as stacked bars for each model. The figure reinforces the performance hierarchy quantified in Table 1 while visually surfacing two distinct patterns. The first is a high-pass, low-equivocal profile exemplified by LLM F (OpenEvidence; 89% Pass, 11% Equivocal) and LLM C (DeepSeek V3.2; 70% Pass, 26% Equivocal), both of which demonstrated predominantly favorable outcomes with minimal Fail ratings. The second is a broader, more dispersed distribution observed for LLM D (Gemini 3 Pro) and LLM E (Grok 4.1), where only 44% of ratings reached the Pass threshold, 41% were Equivocal, and 15% were classified as Fail, indicating that for these models, graders were frequently uncertain about or dissatisfied with the adequacy of clinical reasoning. LLM A (ChatGPT 5.1) exhibited a distinctive pattern: a moderate Pass rate (56%) accompanied by a high equivocal proportion (41%), suggesting a tendency toward safe but incomplete answers that meet minimum acceptability standards without demonstrating the clinical specificity expected of a board-certified surgeon. LLM B (Sonnet 4.5) similarly achieved a 56% Pass rate, but its 15% Fail rate, compared to LLM A's 3%, indicates a greater frequency of substantive reasoning deficits when errors did occur. The complete absence of Critical Fail ratings across all models and all graders is noteworthy and consistent with the interpretation that frontier LLMs anchor toward conservative, safe-harbor responses in clinical reasoning contexts. 

**Figure 2:**
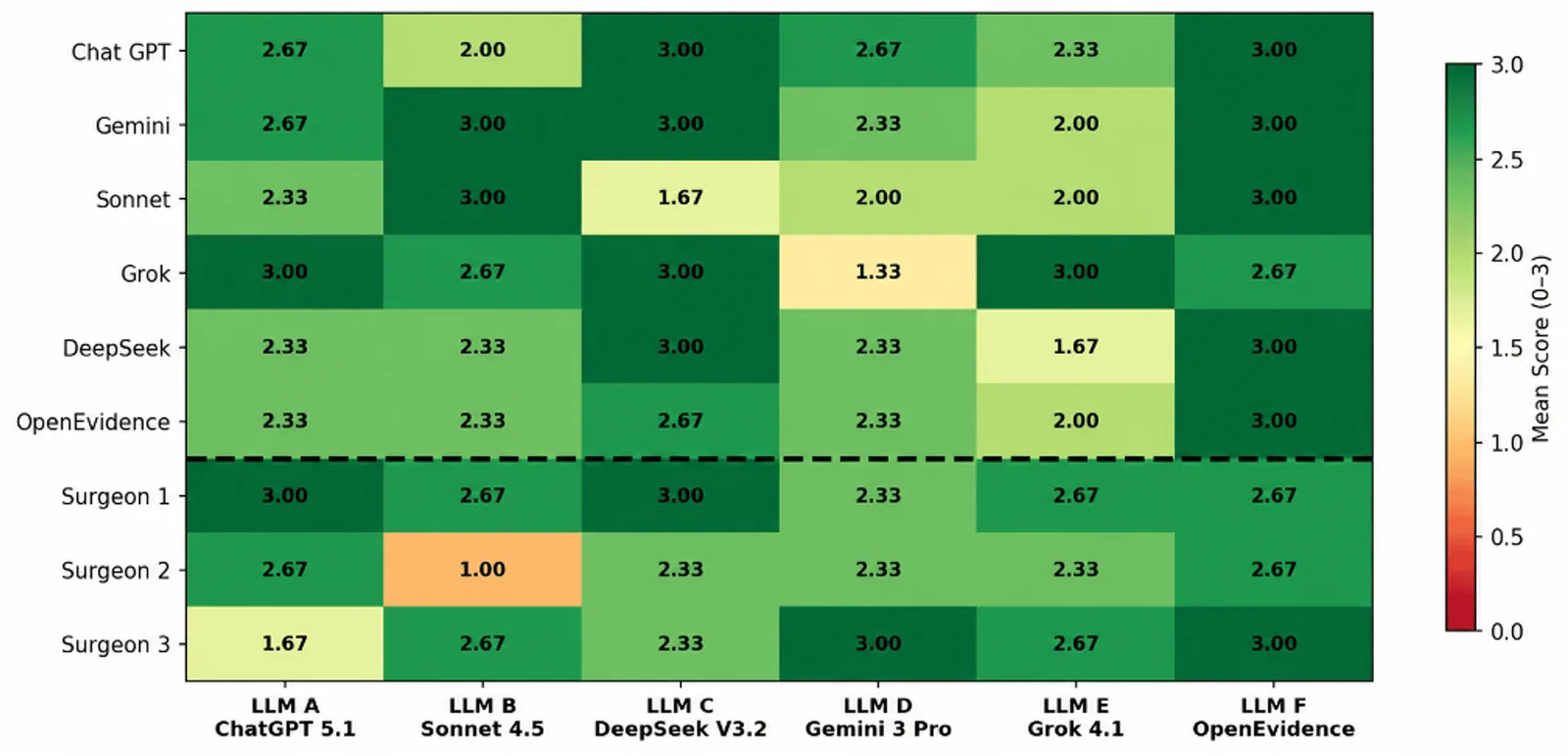
Mean score heatmap of graders and test-takers Heatmap displaying the mean score assigned by each of nine graders (rows) to each of six large language models (LLM) examinees (columns), averaged across all three clinical cases. Graders are organized into two groups separated by a dashed horizontal line: AI graders (upper six rows: ChatGPT, Gemini, Sonnet, Grok, DeepSeek, OpenEvidence) and human surgical graders (lower three rows: Surgeons 1-3). Columns represent examinee identity, with model names displayed on the x-axis. Cell color encodes mean score intensity on a continuous scale from 0.0 (red; Critical Fail) to 3.0 (dark green; Pass), as indicated by the legend. Intermediate values appear as yellow (approximately 1.5-2.0) and mid-green (approximately 2.0-2.5). ChatGPT 5.1 (OpenAI, Inc., San Francisco, California, USA), Gemini 3 Pro (Google LLC, Mountain View, California, USA), Claude Sonnet 4.5 (Anthropic PBC, San Francisco, California, USA), Grok 4.1 (xAI Corp., San Francisco, California, USA), DeepSeek-V3.2 (DeepSeek, Hangzhou, Zhejiang, China), OpenEvidence (OpenEvidence, Inc., Miami, Florida, USA)

Mean scores disaggregated by grader-examinee pairing are illustrated in Figure 2, which presents a heatmap of average scores across all three cases for every grader-examinee combination. This visualization reveals both the central tendency of each pairing and the degree of scoring heterogeneity across the full panel. Among AI graders (upper portion of the heatmap, above the dashed dividing line), scores were generally clustered between 2.00 and 3.00, with limited extreme values; nearly all AI graders assigned scores of ≥2.33 to LLM C (DeepSeek V3.2) and LLM F (OpenEvidence), reflecting broad consensus on the superior performance of these two examinees. The most notable exception was Grok's assignment of a mean score of 1.33 to LLM D (Gemini 3 Pro), representing the lowest AI-assigned score in the dataset and an outlier relative to the other AI graders' assessments of that same examinee. Among human graders (lower portion of the heatmap, below the dashed line), substantially greater scoring dispersion was observed. The most extreme value in the entire dataset, a mean score of 1.00, was assigned by Surgeon 2 to LLM B (Sonnet 4.5), a score reflecting a consistent pattern of Fail ratings across all three cases for that examinee-grader pairing. Surgeon 3, by contrast, assigned a notably lower score of 1.67 to LLM A (ChatGPT 5.1), while Surgeon 1 and most AI graders rated this model considerably higher. These divergences in rank-ordering, where graders not only differed in stringency but systematically disagreed on which examinees performed better, foreshadow the Examinee × Rater interaction that would emerge as the dominant source of variance in subsequent decomposition analyses. Across all graders, LLM F (OpenEvidence) was the most consistently and highly rated examinee, with all graders assigning mean scores of ≥2.67, providing the clearest evidence of consensus at the top of the performance distribution. 

Grader characteristics

**Figure 3:**
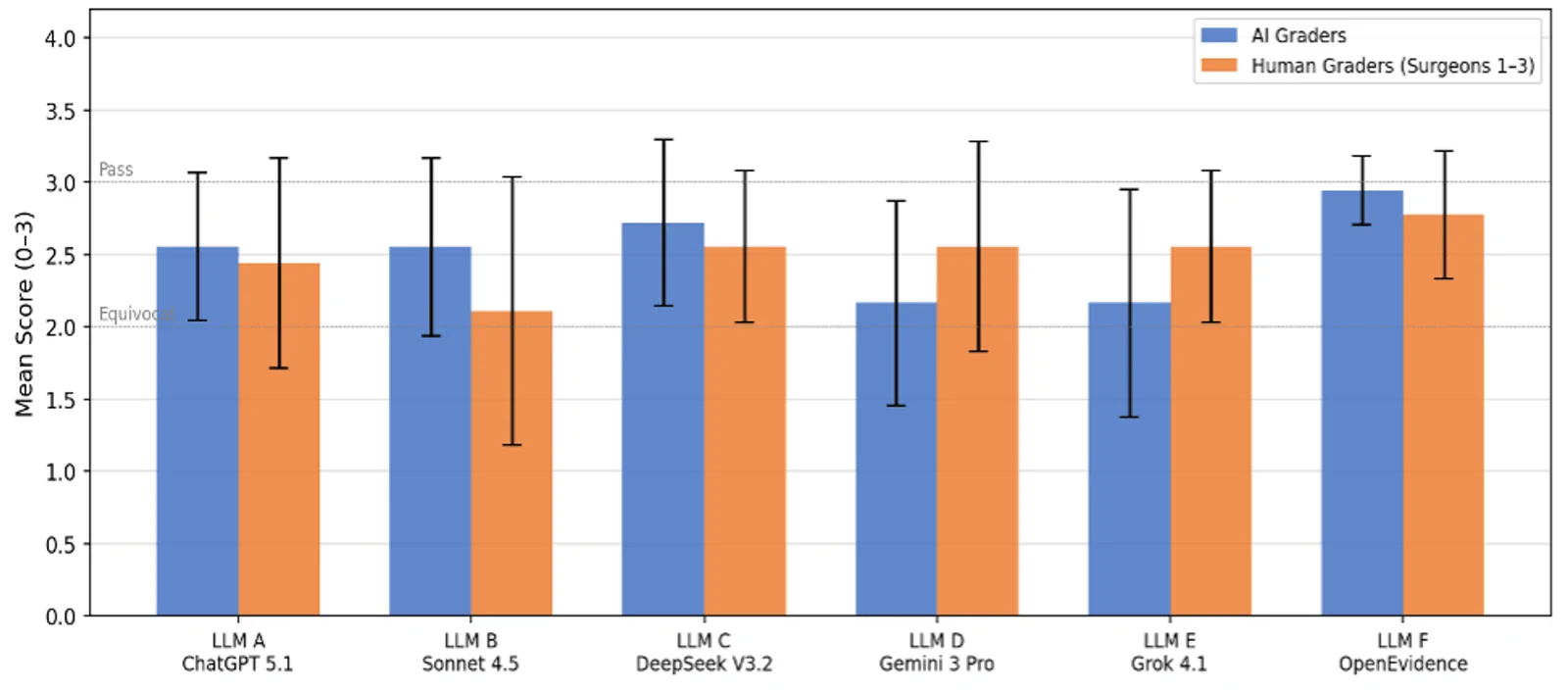
Grader score distribution by examinee and grader type Grouped bar chart comparing mean scores assigned by AI graders (blue bars, n = 6) and human surgical graders (orange bars, n = 3) for each of the six large language models (LLMs) examinees, with error bars representing 95% confidence intervals derived from the within-group distribution of scores across all graders and cases. The y-axis represents the mean score on the 0-3 ordinal scale; horizontal reference lines at y = 2.0 and y = 3.0 mark the Equivocal and Pass thresholds, respectively. ChatGPT 5.1 (OpenAI, Inc., San Francisco, California, USA), Gemini 3 Pro (Google LLC, Mountain View, California, USA), Claude Sonnet 4.5 (Anthropic PBC, San Francisco, California, USA), Grok 4.1 (xAI Corp., San Francisco, California, USA), DeepSeek-V3.2 (DeepSeek, Hangzhou, Zhejiang, China), OpenEvidence (OpenEvidence, Inc., Miami, Florida, USA)

Descriptive statistics for grader scoring behavior are presented in Table 2. Mean grader scores across all examinee-case observations ranged from 2.22 (Surgeon 2) to 2.72 (Surgeon 1), with a grand mean of 2.51. The leniency index, defined as each grader's mean score minus the grand mean, ranged from +0.21 (Surgeon 1) to −0.29 (Surgeon 2), a spread of 0.50 scale points. Among AI graders, leniency ranged from +0.15 (Gemini) to −0.18 (Sonnet), a substantially narrower spread of 0.33 scale points, indicating that AI graders as a class occupy a more compressed and central leniency distribution than their human counterparts. The four most lenient graders in the cohort were Surgeon 1, Gemini, ChatGPT, and Grok, while the most stringent were Surgeon 2, Sonnet, and DeepSeek/OpenEvidence (tied). Grader score distributions across all examinee-case pairings are visualized in Figure 3, which presents mean scores with 95% confidence intervals for AI graders (blue) and human graders (orange) within each examinee grouping.

**Table 2:** Grader-level scoring characteristics and leniency index Descriptive statistics for all nine retained graders (six artificial intelligence graders and three human surgeons) across all examinee-case observations (n = 18 ratings per grader). Graders are ranked in descending order of mean score (most lenient to most stringent). The leniency index is defined as the difference between each grader's mean score and the overall grand mean (2.51); positive values indicate above-average leniency and negative values indicate above-average stringency. ChatGPT 5.1 (OpenAI, Inc., San Francisco, California, USA), Gemini 3 Pro (Google LLC, Mountain View, California, USA), Claude Sonnet 4.5 (Anthropic PBC, San Francisco, California, USA), Grok 4.1 (xAI Corp., San Francisco, California, USA), DeepSeek-V3.2 (DeepSeek, Hangzhou, Zhejiang, China), OpenEvidence (OpenEvidence, Inc., Miami, Florida, USA)

Grader	Type	Mean	SD	Median	Leniency Index
Surgeon 1	Human	2.72	0.46	3	0.21
Gemini	AI	2.67	0.69	3	0.15
ChatGPT	AI	2.61	0.7	3	0.1
Grok	AI	2.61	0.7	3	0.1
Surgeon 3	Human	2.56	0.62	3	0.04
DeepSeek	AI	2.44	0.62	2.5	−0.07
OpenEvidence	AI	2.44	0.62	2.5	−0.07
Sonnet	AI	2.33	0.59	2	−0.18
Surgeon 2	Human	2.22	0.81	2	−0.29

Comparison of AI and human graders

Mean scores by grader type for each examinee are presented in Table 3. Examined at the aggregate examinee level, AI graders assigned numerically higher mean scores than human graders for four of six examinees (LLM A, LLM B, LLM C, and LLM F), while human graders were numerically higher for LLM D and LLM E. However, none of these directional differences reached statistical significance by the Mann-Whitney U test (all p > 0.05), indicating that AI and human graders are broadly comparable in average stringency when scores are collapsed across cases and graders. The largest absolute difference between AI and human mean scores was observed for LLM B (Sonnet 4.5), where AI graders assigned a mean of 2.56 compared to 2.11 by human surgeons (Δ = 0.45 scale points), though this difference also failed to reach significance (U = 11.0, p = 0.691). The convergence between AI and human graders means that, at the aggregate level, it is itself a substantive finding: it suggests that, while AI and human graders differ profoundly in their internal consistency and rank-ordering behavior, they do not differ systematically in average leniency. This decoupling of mean-level agreement from rank-order agreement has important implications for the interpretation of reliability coefficients, as graders can assign statistically indistinguishable average scores while systematically disagreeing about which examinees performed better or worse.

**Table 3:** Comparison of mean scores assigned by AI versus human graders for each examinee Mean scores and standard deviation (±SD) assigned to each of the six large language models (LLM) examinees, disaggregated by grader type: artificial intelligence (AI) graders (n = 6) and human surgical graders (n = 3), averaged across all three clinical cases. Scores are on the 0-3 ordinal scale. Between-group differences were evaluated using the Mann-Whitney U test; no comparison reached statistical significance at α = 0.05. U: Mann-Whitney U statistic; SD: standard deviation ChatGPT 5.1 (OpenAI, Inc., San Francisco, California, USA), Gemini 3 Pro (Google LLC, Mountain View, California, USA), Claude Sonnet 4.5 (Anthropic PBC, San Francisco, California, USA), Grok 4.1 (xAI Corp., San Francisco, California, USA), DeepSeek-V3.2 (DeepSeek, Hangzhou, Zhejiang, China), OpenEvidence (OpenEvidence, Inc., Miami, Florida, USA)

Label	Identity	AI Mean	AI SD	Human Mean	Human SD	U	p
LLM A	ChatGPT 5.1	2.56	0.25	2.44	0.57	8.5	1
LLM B	Sonnet 4.5	2.56	0.37	2.11	0.79	11	0.691
LLM C	DeepSeek V3.2	2.72	0.49	2.56	0.31	12	0.477
LLM D	Gemini 3 Pro	2.17	0.42	2.56	0.31	5	0.322
LLM E	Grok 4.1	2.17	0.42	2.56	0.16	3.5	0.185
LLM F	OpenEvidence	2.94	0.12	2.78	0.16	13.5	0.209

Variance decomposition

A three-way ANOVA with omega-squared (ω²) effect sizes, applied to the fully crossed examinee-by-rater-by-case design, identified the Examinee × Rater interaction as the dominant source of score variance in the dataset (ω² = 0.230; 36.0% of the total sum of squares (SS)). This interaction term reflects systematic disagreement among graders in the rank-ordering of examinees, a pattern qualitatively distinct from simple leniency differences (rater main effect), which were themselves modest (ω² = 0.031; 5.6% of SS). Although the four-point rating scale is technically ordinal, the application of ANOVA for variance decomposition is justified by a well-established body of psychometric literature demonstrating that parametric tests of point estimates, such as ANOVA and t-tests, exhibit robustness to violations of the interval-scaling assumption for Likert-type response formats with multiple ordered categories [14,15]. Critically, the examinee main effect, which represents the variance directly attributable to genuine differences in examinee performance, was small-to-moderate (ω² = 0.090; 10.7% of SS), indicating that actual performance differences among the six LLMs accounted for a substantially smaller share of total score variability than grader-driven disagreement. Case difficulty contributed negligibly to variance (ω² ≈ 0.000), suggesting that the three chosen cases did not differentially discriminate among examinees and were broadly comparable in difficulty. The Rater × Case interaction was non-trivially large (ω² = 0.104; 15.6% of SS), indicating that different graders responded differently to the same cases, some finding certain cases more discriminating than others, adding a second layer of grader-driven variability beyond simple leniency differences. The residual term (unexplained variance) accounted for 26.0% of SS, consistent with a measurement context in which a substantial proportion of individual score variation cannot be attributed to any systematic source and likely reflects idiosyncratic case-by-grader-by-examinee interactions. Full decomposition results are presented in Table 4. These findings converge on a structural diagnosis of the current assessment framework: the dominant determinant of oral board scores is not what an examinee knows, but who is grading them, a conclusion with direct implications for the validity and equity of pass/fail decisions made on the basis of this instrument. 

**Table 4:** Variance decomposition from three-way ANOVA (Examinee × Rater × Case) Results of a fully crossed three-way analysis of variance decomposing total score variance into systematic and residual components across the examinee, rater, and case dimensions. The dataset consisted of 162 observations (six examinees × nine graders × three cases). Effect sizes are reported as eta-squared (η²; unadjusted proportion of total SS) and omega-squared (ω²; bias-corrected population variance estimate). SS: sum of squares; MS: mean square; df: degrees of freedom

Source	SS	df	MS	η²	ω²
Examinee	7.29	5	1.458	0.107	0.09
Rater	3.864	8	0.483	0.056	0.031
Case	0.086	2	0.043	0.001	0
Examinee×Rater	24.654	40	0.616	0.36	0.23
Examinee×Case	4.21	10	0.421	0.062	0.029
Rater×Case	10.691	16	0.668	0.156	0.104
Residual	17.679	80	0.221	None	None

Internal consistency

Internal consistency metrics revealed a profound divergence in Cronbach's α between AI and human grader panels. The AI-only panel (n = 6 graders, k = 6) achieved α = 0.697, approaching the conventional adequacy threshold of 0.70 for multi-rater assessment panels and representing the highest α of any panel configuration tested. The Spearman-Brown projection for a doubled AI panel (2k = 12 graders) yields a projected α of 0.821, exceeding the 0.80 benchmark, and the minimum panel size required to reach α ≥ 0.80 was estimated at eight AI graders, indicating that a modestly expanded AI grader panel could achieve acceptable reliability for high-stakes decision-making. By contrast, the human surgeon panel (n = 3, k = 3) produced a Cronbach's α of −0.923, a value that cannot be interpreted as low reliability but rather signals systematic anti-correlation among raters; because the Spearman-Brown formula is undefined for negative α, no doubled-panel projection or minimum-rater estimate could be calculated for this panel. A negative α occurs when the average inter-item covariance is negative, meaning that graders are not simply noisy but are actively rank-ordering examinees in opposing directions. This finding is consistent with divergent implicit scoring standards among senior surgeons, though the small panel size (n = 3) precludes definitive conclusions; a negative Cronbach's α with three raters can arise from sample size constraints alone, and replication with a larger human panel is needed before strong interpretive claims are warranted. The full nine-grader cohort (k = 9) produced α = 0.577, which falls in the "poor" range by the same interpretive bands (0.50-0.59); the Spearman-Brown projection for a doubled cohort (2k = 18 graders) was 0.732, and the minimum panel size required to reach α ≥ 0.80 was estimated at 12 total graders. Item-total correlations further revealed that OpenEvidence was the sole grader to demonstrate a statistically significant positive correlation with the overall panel consensus score (r = 0.880, p = 0.021), identifying it as the single most panel-consistent grader in the dataset. Multiple graders, including Grok (AI) and Surgeon 3, demonstrated near-zero or negative item-total correlations, indicating that their individual scores were not systematically aligned with the aggregate panel judgment.

Decision consistency

Pass/fail decision consistency metrics across grader panel configurations are presented in Table 5. When translated into binary pass/fail outcomes, inter-grader agreement was uniformly modest across all panel configurations. Mean pairwise agreement was 0.556 (95% CI (0.52, 0.59)) for the full nine-grader cohort, 0.619 (95% CI (0.56, 0.67)) for the AI-only panel, and 0.519 (95% CI (0.44, 0.61)) for the human surgeon panel. The non-overlapping confidence intervals for the AI-only and human panels suggest a reliable, if modest, advantage in agreement for AI graders, though all panels remained well below the levels expected of a high-stakes certification instrument. 

**Table 5:** Pass/fail decision consistency across grader panel configurations Pairwise pass/fail agreement rates and the proportion of examinee-case combinations yielding mixed decisions (at least one grader disagreeing with peers) across four panel configurations. Pass/fail decisions were derived by dichotomizing ordinal scores at a threshold of ≥ 2 (Equivocal or Pass) versus ≤ 1 (Fail). This threshold was chosen to reflect a conservative decision boundary. Pairwise agreement was calculated as the proportion of examinee-case pairings on which two graders rendered identical pass/fail verdicts, averaged across all unique grader pairs within each panel. Mixed cells refer to the 18 unique examinee-case combinations (six examinees × three cases) in which at least one grader's pass/fail decision conflicted with at least one peer. A 95% confidence interval (CI) derived by bootstrapping over grader pairs (2,000 iterations).

Panel	Grader Pairs	Mean Agreement	95% CI	Min	Max	Mixed Cells
Full cohort (n = 9)	36	0.556	(0.52, 0.59)	0.389	0.778	17/18 (94.4%)
AI graders only (n = 6)	15	0.619	(0.56, 0.67)	0.444	0.778	15/18 (83.3%)
Human graders 1-3 (n = 3)	3	0.519	(0.44, 0.61)	0.444	0.611	13/18 (72.2%)

Self-grading sensitivity analysis

A sensitivity analysis excluding self-grading observations (each AI model's rating of its own responses) was conducted to assess whether self-serving bias inflated AI grader reliability or examinee performance estimates. Five of six AI models assigned themselves numerically higher scores than they received from peer graders, a consistent directional pattern suggesting a modest tendency toward self-favorable evaluation. However, this directional effect did not reach statistical significance (t = 2.02, p = 0.10), and the overall magnitude of self-inflation was small relative to the observed between-grader variance. Gemini was the sole exception, assigning itself a lower score than its peer average, a finding inconsistent with a systematic self-serving mechanism and suggestive of model-specific evaluation conservatism. These findings indicate that self-grading bias, while present directionally, does not substantially alter the primary psychometric conclusions of the study. Nevertheless, given the non-trivial directional effect and the high-stakes context of board examination scoring, exclusion of self-grading observations from panel-aggregated scores is recommended as a precautionary design principle in any AI-augmented assessment framework where models function as both examinees and graders simultaneously. A score matrix of raw data from models and surgeon reviewers that was used to calculate all analyses is provided in Appendix 1.

## Discussion

Principal findings

This study addresses two foundational questions regarding the potential integration of LLMs into surgical board certification assessment: whether contemporary LLMs can demonstrate reasoning consistent with passing oral board performance, and whether AI graders exhibit comparable or superior reliability relative to trained human surgeons. The results suggest an affirmative answer to both questions, with important caveats. All six contemporary LLMs achieved at minimum equivocal-to-passing performance across standardized general surgery oral board-style cases, and no Critical Fail ratings were recorded across 162 graded observations, indicating that modern models have internalized sufficient clinical knowledge to avoid catastrophically unsafe surgical recommendations without task-specific fine-tuning in this simulation context. AI graders demonstrated substantially greater internal consistency relative to a panel of senior human surgeons, though the small human panel size limits the precision of this comparison. The dominant source of score variance was the Examinee × Rater interaction, a structural finding indicating that grader-driven disagreement accounted for more score variability than actual examinee performance differences in this dataset. These findings offer a preliminary empirical foundation for investigating AI integration into surgical assessment pipelines, while also highlighting measurement challenges that warrant further study. 

LLM performance as examinees

The finding that all six tested LLMs achieved at least equivocal competency on general surgery oral board-style cases is notable given the cognitive demands of the format. Unlike multiple-choice licensing examinations, oral board assessment requires examinees to prioritize diagnostic workup, adapt to evolving clinical information, and justify clinical and operative decision-making in real time, reflecting higher levels of Miller’s framework of clinical competence that extend beyond knowledge-based selection among pre-defined answer choices [16]. The performance of state-of-the-art LLMs at this level, without domain-specific fine-tuning, extends a growing body of evidence that these systems have internalized medical knowledge sufficient for basic clinical reasoning tasks [3,4].

OpenEvidence (LLM F) emerged as a consistently high-performing in this 2026 cross-sectional snapshot, achieving near-Pass mean scores from both AI and human graders, recording zero Fail ratings, and demonstrating the narrowest score distribution of any examinee. This model's clinical orientation, purpose-built for evidence-based medical decision support and trained on a curated corpus of peer-reviewed literature, may partially explain its superior and more consistent performance, consistent with prior findings that domain-specific pretraining and instruction tuning improve performance on medically oriented tasks compared to general-purpose models [9]. The performance gap between OpenEvidence and the lowest-scoring models (LLM D, Gemini 3 Pro; LLM E, Grok 4.1) aligns with prior literature suggesting that clinically oriented training and domain-specific augmentation may be associated with improved performance in surgical reasoning tasks compared with general-purpose conversational models [4,5]. More broadly, however, the primary contribution of this work is not a durable ranking of specific model versions, which will inevitably shift as the field progresses, but rather the demonstration that contemporary LLMs, across diverse architectural families, can achieve a minimum competency threshold in this demanding clinical format, and the introduction of a crossed psychometric evaluation framework that can be redeployed to assess future generations of models as they emerge.

The overall fail rate of 8.6%, and up to 14.8% for the lowest-performing models, indicates that current contemporary LLMs are not uniformly reliable substitutes for surgical trainees in high-stakes assessment contexts and that residual failure modes remain clinically meaningful. However, the complete absence of Critical Fail ratings across all models and all graders carries independent significance. While LLMs may produce incomplete or suboptimal answers, they did not generate the catastrophically unsafe clinical recommendations most concerning in a surgical training context, such as recommending non-operative management for a perforated viscus or failing to recognize a surgical emergency. This behavioral pattern is consistent with emerging evidence that clinically aligned LLMs may preferentially generate guideline-consistent and lower-risk recommendations in uncertain clinical scenarios, reflecting safety-oriented instruction tuning that can enhance reliability in medical question answering while potentially limiting granular clinical nuance [17]. Performance variation across models likely reflects differences in training data composition and domain adaptation, and the observation that all models performed above the critical failure threshold suggests a non-zero baseline capability of mainstream LLMs on structured general surgery oral board reasoning tasks. This finding is consistent with prior work demonstrating that LLMs can encode clinically relevant knowledge and perform medical question-answering tasks without domain-specific fine-tuning [9].

AI versus human grader reliability

The greater internal consistency of AI graders relative to the human surgeon panel is among the more striking observations of this study, though it must be interpreted with caution, given the small human panel size. Cronbach's α for the AI-only panel (0.697) approached the conventional adequacy threshold and is projected to exceed the high-stakes reliability benchmark of 0.80 with only eight total AI graders, a panel size readily achievable in practice. The human-only panel produced α = −0.923, a value that cannot be interpreted as simply poor agreement. A negative Cronbach's alpha arises when the average inter-item covariance is negative, meaning that the three surgeons not only disagreed in average stringency but actively rank-ordered examinees in opposing directions. This finding is consistent with prior psychometric analyses documenting that experienced clinicians frequently apply divergent implicit standards even when provided with nominally identical scoring rubrics [2,6]. Qualitative investigation of assessor cognition has identified multiple mechanisms that contribute to variability in performance assessment, including differential salience, criterion uncertainty, and variable information integration, whereby assessors attend to different aspects of performance and construct idiosyncratic evaluative frameworks [18]. These mechanisms highlight that agreement in mean scoring does not necessarily reflect shared underlying judgment processes. The divergence between AI and human grader consistency observed in the present study, despite statistically indistinguishable mean-level leniency, illustrates a critical psychometric distinction: aggregate score equivalence does not imply equivalence in rank-order agreement, and graders who assign similar average scores may nevertheless differ substantially in their relative evaluation of examinees [10,18]. This AI advantage must be interpreted with caution, however, given a key methodological asymmetry: AI graders received the rubric explicitly reinforced within the prompt on every instance, whereas human graders, though provided the same rubric, underwent no formal calibration training. The greater reliability of the AI panel may thus reflect consistent rubric application enforced by prompt engineering rather than intrinsic superiority over human judgment. In the absence of a calibrated human comparator, we refrain from concluding that AI graders are more reliable than humans; rather, our findings demonstrate that AI can achieve acceptable consistency under standardized rubric delivery while underscoring the critical importance of rater calibration for human panels in high-stakes assessment.

The variance decomposition analysis provides a structural context for this finding. The dominance of rater-interaction variance over the examinee main effect indicates that score variability in this assessment was driven primarily by grader disagreement rather than true performance differences, a structural finding with direct implications for the fairness and validity of oral board pass/fail decisions. Overall, the dominance of rater-related variance components over examinee variance is consistent with generalizability theory analyses of high-stakes clinical performance assessments, which frequently identify rater and interaction effects as major sources of measurement error [12,19].

Generalizability theory D-study projections further contextualize this finding. A systematic review and meta-analysis of G-theory applications in surgical and medical skill assessment reported a pooled generalizability coefficient of approximately 0.65 across studies, with many investigations demonstrating that achieving reliability thresholds near 0.80 typically requires optimization of rater and case sampling through D-study design adjustments [19]. These findings suggest that rater and rater-related interaction variance commonly represent major constraints on reliability in performance-based clinical assessments. This is a structural limitation of the current instrument design, not merely a sample size problem. The pass/fail decision consistency analysis reinforces this conclusion at its most operationally consequential level: across 94.4% of all examinee-case combinations, at least one grader rendered a pass/fail verdict that contradicted one or more peers. Under these conditions, binary certification outcomes in oral board examinations may be substantially influenced by examiner composition effects rather than examinee performance alone, a phenomenon consistent with prior work demonstrating significant inter-examiner variability in oral clinical assessments [2,6]. These findings suggest that the reliability of pass/fail decisions in current oral board formats may merit further empirical scrutiny, particularly as the field considers how to incorporate structured feedback mechanisms and standardized assessment tools. 

Implications for surgical education and assessment design

These results have direct and actionable implications for how surgical training programs might incorporate AI into oral board preparation and high-stakes assessment. In the near term, LLMs are well-positioned to serve as consistent, available, and informative practice partners for surgical trainees, providing immediate structured feedback without the scheduling constraints, rater variability, or resource demands of human examiner panels.

In a more transformative framing, clinically oriented AI graders, particularly models such as OpenEvidence, which demonstrated both high performance as an examinee and the strongest panel-consistent scoring as a grader, could be incorporated into structured panel grading designs to anchor consistency and reduce human-driven variance. Hybrid panel configurations combining AI graders with human surgeons may offer the optimal near-term balance between psychometric reliability and clinical face validity, potentially preserving the irreplaceable contribution of human clinical judgment while using AI graders as calibrating anchors. Such a design is analogous to the role of standardized anchor rubrics in other high-stakes medical assessments, where structured scoring criteria have been shown to reduce examiner variability without eliminating human judgment [20].

Future directions

Future work should extend this framework to audio-based AI agent interactions to better replicate the adaptive, real-time format of live oral board examinations, expand the case library across the full scope of general surgery, and leverage ABS educational resources for rubric refinement and model training. Hybrid panel designs combining AI graders with human surgeons warrant prospective evaluation as a mechanism for anchoring grader consistency while preserving clinical judgment. 

Limitations

Several limitations of this study warrant acknowledgment. First, the sample of three cases and six LLMs, while sufficient for proof-of-concept psychometric analysis, limits the generalizability of performance rankings across the full breadth of general surgery topics evaluated on ABS oral examinations. A larger case bank spanning trauma, endocrine, colorectal, vascular, and oncologic surgery would be required to establish stable performance estimates across the full domain. Second, all interactions were text-based and delivered as structured prompts; the adaptive, branching follow-up questioning characteristic of live oral board examinations was approximated but not fully replicated. Human examiners can pursue unexpected reasoning threads, challenge inconsistencies, and probe the depth of knowledge in ways that were not captured by the fixed-prompt design used here. Third, the scoring rubric, while clinician-reviewed and anchored to the 0-3 ordinal scale, was not formally validated against existing ABS scoring instruments, limiting claims of criterion validity relative to the actual certification examination. Fourth, LLM model versions are subject to rapid iterative updates, and the specific performance profiles observed here may not persist across future model generations; the field is evolving at a pace that outstrips the conventional publication cycle [21]. Fifth, the human grader panel was small (n = 3), limiting the precision of human reliability estimates and reducing statistical power for human-only comparisons; a larger human panel would yield more stable Cronbach's alpha estimates and narrower confidence intervals for between-group comparisons. Sixth, this study did not assess examinee responses for granular factual accuracy; only aggregate ordinal pass/fail judgments were analyzed, which may obscure clinically important differences in the quality, specificity, or mechanistic accuracy of model reasoning that are not captured by holistic ordinal ratings. Seventh, all six LLMs evaluated in this study were trained on large, internet-derived corpora that likely include medical board examination preparation materials, case-based learning resources, and clinical vignettes structurally similar to those used here. It is therefore not possible to determine from these results whether observed performance reflects generalizable clinical reasoning or sophisticated pattern-matching on training data; this distinction is critical for interpreting the educational validity of these findings. Eighth, because the fully crossed design permitted each AI model to grade its own responses, a potential for self-serving bias exists despite the sensitivity analysis suggesting this effect was modest. Future study designs should consider excluding self-grading pairings by design rather than by post-hoc sensitivity analysis.

## Conclusions

This study introduces a crossed psychometric framework for evaluating LLMs in performance-based surgical assessment, demonstrating that contemporary frontier models can achieve at least equivocal-to-passing performance on standardized oral board-style cases in simulation, with no Critical Fail ratings recorded across 162 observations. The dominant source of score variance was the Examinee × Rater interaction, indicating that grader-driven disagreement accounted for more variability than examinee performance differences, a finding that underscores the value of variance decomposition as a reusable methodological tool for future evaluations. AI graders demonstrated greater internal consistency than the uncalibrated three-surgeon human panel, though this comparison is limited by the small human sample and the absence of formal rater calibration for human graders; accordingly, we do not interpret this as evidence of intrinsic AI superiority over trained human examiners. These preliminary findings suggest potential value for LLM integration into surgical board preparation as scalable practice partners, while also highlighting rubric standardization and examiner calibration as high-priority targets for improving oral board reliability. Future work should extend this framework to incorporate audio-based AI agent interactions, broaden the case library across the full scope of general surgery, and include comparisons against calibrated human rater panels across multiple model generations.

## Appendices

Appendix 1 

**Table 6:** Raw score matrix Complete raw score matrix (n = 162 observations: six examinee models × nine graders × three cases) underlying the variance decomposition in Table 4 and the internal consistency analysis in Table 5. Each cell is a single grader's score for one examinee model on one case, recorded on a 4-point scale: 0 = Critical Fail, 1 = Fail, 2 = Equivocal, 3 = Pass. AI: artificial intelligence; LLM: large language model; OE: OpenEvidence The six AI graders (ChatGPT, Gemini, Sonnet, Grok, DeepSeek, OE) are the same six large language models evaluated as examinees, here acting in the grader role; this includes instances of self-grading (e.g., ChatGPT grading the ChatGPT 5.1 examinee transcript). Surgeon 1, Surgeon 2, and Surgeon 3 are the three human general surgery attendings retained in the primary analysis. n = number of graders in a given panel; k = number of items (graders) used in reliability calculations. A fourth human grader was initially recruited but excluded from the primary analysis due to incomplete data submission; this individual did not provide ratings for all examinees across all cases, and their partial data could not be incorporated into the fully crossed design.

Examinee Model	Case	ChatGPT (AI)	Gemini (AI)	Sonnet (AI)	Grok (AI)	DeepSeek (AI)	OE (AI)	Surgeon 1	Surgeon 2	Surgeon 3
ChatGPT 5.1	1	3	3	3	3	3	3	3	3	2
ChatGPT 5.1	2	3	2	2	3	2	2	3	2	2
ChatGPT 5.1	3	2	3	2	3	2	2	3	3	1
Claude Sonnet 4.5	1	2	3	3	2	3	2	2	1	2
Claude Sonnet 4.5	2	3	3	3	3	2	3	3	1	3
Claude Sonnet 4.5	3	1	3	3	3	2	2	3	1	3
DeepSeek V3.2	1	3	3	1	3	3	2	3	2	3
DeepSeek V3.2	2	3	3	2	3	3	3	3	2	2
DeepSeek V3.2	3	3	3	2	3	3	3	3	3	2
Gemini 3 Pro	1	3	3	2	1	3	3	2	1	3
Gemini 3 Pro	2	3	1	2	1	2	2	2	3	3
Gemini 3 Pro	3	2	3	2	2	2	2	3	3	3
Grok 4.1	1	3	3	2	3	2	2	2	2	3
Grok 4.1	2	3	2	2	3	1	3	3	2	2
Grok 4.1	3	1	1	2	3	2	1	3	3	3
OpenEvidence	1	3	3	3	3	3	3	2	2	3
OpenEvidence	2	3	3	3	3	3	3	3	3	3
OpenEvidence	3	3	3	3	2	3	3	3	3	3
